# The Role of Antibiotic Prophylaxis in Reducing Bacterial Contamination of Autologous Bone Graft Collected from Implant Site

**DOI:** 10.1155/2017/2175019

**Published:** 2017-12-20

**Authors:** Rodolfo Mauceri, Giuseppina Campisi, Domenica Matranga, Nicola Mauceri, Giuseppe Pizzo, Dario Melilli

**Affiliations:** ^1^Department of Surgical, Oncological and Oral Sciences, University of Palermo, Palermo, Italy; ^2^Department of Health Promotion Sciences and Mother-Child Care “G. D'Alessandro”, University of Palermo, Palermo, Italy

## Abstract

The aim of this study was to evaluate if antibiotic prophylaxis reduces the bacterial contamination of bone particles collected directly from the burs used for implant site preparation. Thirty-four patients underwent the surgical procedures for a total of 34 implant sites. One 1 gr. tablet of amoxicillin + clavulanic acid was given to the test group 12 hours and 1 hour before the surgery. The control group did not take antibiotic prophylaxis. Bone particles were collected and centrifuged. The suspensions were subjected to serial dilutions and each dilution was examined twice using a spatulation technique in Trypticase Soy Agar (TSA), in Sabouraud Dextrose Agar, and in Mitis Salivarius Agar (MSA). The number of colonies was calculated and the identification of various microorganisms was made. The most represented species, in both groups of patients, belonged to the “oral Streptococci.” For TSA, the test and control groups differed significantly (*p* = 0.018). Conversely, there was no significant difference for MSA (*p* = 0.201) and for the number of bacterial species isolated in the samples of the two groups of patients (*p* = 0.898). The antibiotic prophylaxis reduced, but did not cancel, the risk of infection of the autogenous particulate bone graft. This trial is registered with IRCT2017102537002N1.

## 1. Introduction

An important premise for the long-term success of implant supported prosthetic restoration, including implant overdentures, is a proper implant placement in a sufficient volume of healthy bone recipient [1, 2]. The implant surface and sufficient amount of bone in the implants beds are currently considered two of the most important factors for osseointegration [3, 4]. During the surgical placement of the implant fixtures, peri-implant defects such as dehiscences or fenestrations can occur; this may be due to the bone anatomy of an edentulous area, where bone volume is frequently lacking as a result of trauma, tooth loss, or infection diseases such as chronic periodontitis. In all of these situations, bone regeneration of the defect would improve the long-term prognosis for the implant [3, 5, 6].

Among graft materials currently used to correct bone defects, autologous bone is described as the gold standard one, as it has biocompatibility, osteoinductive and osteoconductive properties, without immunological reactions, and extra cost for purchasing biomaterials [3, 5, 7, 8]. In the presence of small bone defects, the bone particles produced during the preparation of the implant site could be used to cover the exposed implant surface [3, 5]. Therefore, a valuable surgical protocol should contemplate collecting bone particles during the implant site preparation [9].

Worthy of note, the bacterial contamination of collected bone may occur, due to the high bacterial charge of the saliva that reaches values of 10^9^ CFU/mL [10–13]. In bone particles collected during dental implant surgery, pathogen bacterial species, such as* Enterococcus faecalis* and* Staphylococcus epidermidis*, have been isolated [10, 11]. It has also been suggested that bacterial contamination of the bone debris may be a cause of failure of osseointegration of the implants, due to the increased local inflammatory response that compromises the healing of the bone [14]. A number of studies have suggested different methods to reduce bacterial contamination of the collected bone: antibiotic prophylaxis, disinfection of the oral mucosa before the surgery, use of specific bone collectors, stringent aspiration protocol, or surgical techniques [10, 11, 15–17]. Among these, antibiotic prophylaxis has been suggested as useful when collecting bone particles for bone augmentation [16, 18]. Nevertheless, the role of antibiotic prophylaxis to reduce bacterial contamination of bone particles is still controversial, due to the small number of heterogeneous studies published on the topic.

The aim of this study was to evaluate if a protocol of antibiotic prophylaxis reduces the physiologically occurring bacterial contamination of the bone particles collected directly from the burs used for preparation of the implant site.

## 2. Materials and Methods

This study protocol conformed to the ethical guidelines of the 1964 Declaration of Helsinki and its later amendments or comparable ethical standards and was approved by institutional review board of the University of Palermo General Hospital (AOU Policlinico Paolo Giaccone; approval number 8/2014). The study was registered at Iranian Registry of Clinical Trials (IRCT2017102537002N1).

Eligibility criteria for participants and setting were as follows: a need to replace the absence of one tooth with an implant supported prosthesis, no systemic contraindications to surgical procedures, no pregnancy, no history of antibiotic therapy for 6 months prior to the study, no allergy to penicillin or related drugs, absence of carious lesions and local active inflammatory conditions or chronic/aggressive periodontitis, and presence of alveolar ridges of class II or III of Cawood and Howell [19]. Exclusion criteria included systemic diseases that affect jaws and periodontium, orthodontic appliance, active dental caries, chronic diseases of the oral mucosa, chronic antibiotic usage, antiplaque mouthrinse consumption, drugs consumption which affect saliva flow rate, and radiotherapy history.

In the period between September 2014 and February 2015, 34 patients (14 men and 20 women, mean age: 49.7) were selected and underwent the same surgical procedures, for a total of 34 implant sites. All the procedures were performed at the University of Palermo General Hospital; after being informed, patients signed written consent for both surgical treatment and the experimental study.

Fifteen days before the surgery, all patients were subjected to an oral hygiene session and instructed to perform correct domiciliary oral hygiene, improved with 0.2% chlorhexidine mouthrinses (Meridol Clorexidina, Colgate-Palmolive, Roma, Italy) after tooth brushing twice a day.

Patients were randomly divided into two groups using a predetermined computer-generated randomization scheme. Antibiotic prophylaxis was prescribed to the test group, consisting of 18 patients, while the control group of 16 patients did not take antibiotic prophylaxis at all. One 1 gr. tablet of amoxicillin + clavulanic acid (Augmentin, GlaxoSmithKline, Verona, Italy) was given to the test group patients 12 hours and 1 hour before the surgery.

All surgical procedures were performed by the same operator. Both the surgeon and assistants wore sterile gowns and gloves, while the patients were fully covered with sterile drapes in the usual manner. The lips and perioral facial skin areas of the patients were disinfected with benzalkonium chloride. Before surgery all the patients were asked to rinse their mouth for 60 seconds with a chlorhexidine 0.2% mouthrinse. After local anesthesia, a full-thickness flap was made and the implant site was prepared with calibrated drills (Intra-Lock International, Boca Raton, FL, USA) at 600 revolutions per minute, irrigated by cooled saline solution. During each step of the calibrated drills, bone particles on the coils were collected and placed in a 15 ml sterile tube containing 10 ml of prereduced thioglycolate broth (Oxoid, Cambridge, UK).

All implants were submerged, and they were exposed 3 months after implant placement, in order to complete the implant-prosthetic treatment. Follow-up visits were scheduled at 1, 3, 6, and 12 months.

The collected samples were sent to the microbiology laboratory within one hour. The tubes containing the bone particles were centrifuged at 4000 ×g for 10 minutes at 4°C. Under a laminar flow hood, the supernatant was removed from the tubes and the bone particles, after being weighed, were resuspended in 2 ml of sterile PBS solution at pH 7.4 and transferred to a new sterile tube. The suspensions were subjected to serial dilutions (from 10^−1^ to 10^−4^) and 100 *μ*l of each dilution was examined twice, using the spatulation technique, in Trypticase Soy Agar (TSA) for total counts of microorganisms, in Sabouraud Dextrose Agar (SDA) for yeast-form cell counts, and in Mitis Salivarius Agar (MSA) for streptococci isolation. TSA and SDA plates were incubated at 37 ± 1°C for 48 h in aerobic conditions; MSA plates were placed in an incubator within a controlled atmosphere, at 5% CO_2_ and 37°C ± 1 for 96 hours.

The number of colonies present on the different culture medium was multiplied by the dilution factor of the sample. The numbers of colony forming units per bone samples (CFU/g) were calculated according to Kürkçü et al. [16].

The identification of the various microorganisms on pure cultures, obtained from subtransplants in nonselective media (diagnostic sensitivity test agar, DSTA), was made using morphological criteria, microscopic observation after Gram staining, and biochemical tests: catalase, coagulase, and API Identification Systems (bioMérieux, Marcy l'Etoile, France). Moreover, the Blood Agar Medium was used for the Gram-positive and catalase negative colonies to emphasize the presence and type of hemolysis.

### 2.1. Statistical Analysis

Abnormally distributed quantitative variables were log-transformed and summarized as median and interquartile range (IR). Counts and proportions were calculated for qualitative variables. Frequencies were analyzed using Fischer's exact test whereas medians were compared using the Wilcoxon rank-sum test. Data were analyzed using Stata/SE 14.1 (Stata Corporation, College Station, TX, USA). Statistical significance was defined as *p* ≤ 0.05, two-tailed.

## 3. Results

The test and control groups did not differ significantly for age and clinical variables (Table 1). All the implants placed successfully complete the osseointegration process, without any complications during healing period. No prosthesis failed during the entire follow-up, and no major complications were recorded.

**Figure 1:**
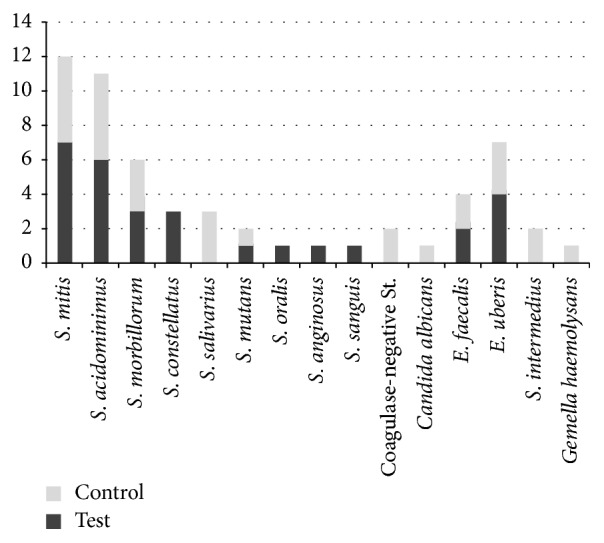
Bacterial species isolated in the samples of the two groups of patients.

The most represented species, in both groups of patients, belonged to the group of bacteria called “oral streptococci.” The most frequently isolated microorganisms in both groups are listed in Table 2 and Figure 1:* Streptococcus mitis* (test group 38.8%; control group 31.2%),* Streptococcus acidominimus *(test group 33.3%; control group 31.2%),* Streptococcus uberis *(test group 22.2%; control group 18.7%), and* Streptococcus morbillorum* (test group 16.6%; control group 18.7%). Other microorganisms found in both test and control groups were* Streptococcus mutans* (test group 5.5%; control group 6.25%) and* Enterococcus faecalis* (test group 11.1%; control group 12.5%).* Streptococcus oralis* (5.5%),* Streptococcus sanguis* (5.5%), and* Streptococcus anginosus* (5.5%) were found only in the test group, whereas* Streptococcus salivarius* (18.7%),* coagulase-negative Staphylococcus* (12.5%),* Streptococcus intermedius* (12.5%),* Gemella haemolysans* (6.25%), and* Candida albicans* (6.25%) were found only in the control group.

The median (IR) values of the test group were 1.71 (0.78–1.91) in the TSA and 1.03 (0.60–1.78) in the MSA, whereas in the control group they were 2.12 (1.15–3.42) and 1.62 (0.82–3.03), respectively. For TSA, the test and control groups differed significantly (*p* = 0.018). Conversely, there was no significant difference for MSA (*p* = 0.201) and for the number of bacterial species isolated in the samples of the two groups of patients (*p* = 0.898) (Table 3).

## 4. Discussion

Oral surgical procedures are often graded as class II (clean-contaminated surgery), with a rate of local infection of 10–15% [20, 21]. This rate could be higher in implant surgery, particularly when bone particles harvested in bone collectors are used in presence of exposed implant fixtures (“simultaneous augmentation technique”) [22].

As bone particles are always contaminated by bacteria, their use could promote the formation of a bacterial biofilm at the level of the peri-implant tissues, affecting the result of the bone regeneration technique and reducing the success rate of the implant therapy [10, 16, 23–27].

The use of stringent aspiration protocol and the preoperative rinsing with chlorhexidine mouthrinse has been demonstrated to minimize bacterial contaminants, but none of these methods are able to completely eliminate bacterial contamination of bone particles [10, 11, 15–17]. Conversely, a number of authors recommended that patients should receive prophylactic antibiotic treatment as a routine protocol before undergoing intraoral bone augmentation procedures with bone particles harvested with bone traps [11, 15, 18, 28–34].

In this study, we evaluated the efficacy of an antibiotic prophylaxis regimen (one 1 gr. tablet of amoxicillin plus clavulanic acid 12 hours and 1 hour before the surgery) on the bacterial contamination of the bone particles harvested during the preparation of the implant site. Oral Gram-positive and Gram-negative bacteria are largely susceptible to amoxicillin-clavulanate, and amoxicillin is usually the drug of choice within the beta-lactam family because it is better absorbed, following oral administration, than other beta-lactam antibiotics [35]. All the patients underwent a preoperative oral rinse with 0.2% chlorhexidine, but antibiotic prophylaxis was administered only in the test group. The bone particles were collected from the calibrated burs used for the preparation of the implant site, without the use of bone collectors.

Our results showed bacterial contamination in all the bone particle samples analyzed. Similarly, to the results reported by Young et al. [10], the species detected included both commonly occurring and rare oral isolates. The most frequently isolated bacteria belong to the group of “oral streptococci” (Figure 1). Among these,* Streptococcus constellatus* and* Streptococcus mitis *are considered potential responsible for odontogenic infections.* Streptococcus intermedius* is an anaerobic commensal bacterium, belonging to the* S. anginosus* group. Moreover, despite being commensal organisms, members of the* S. anginosus* group have been isolated from patients with periodontitis [36]. Other microorganisms isolated, not belonging to oral streptococci group, are* Enterococcus faecalis*,* Streptococcus uberis,* and* coagulase-negative Staphylococcus*. Despite the small number of samples, the statistical analysis showed that when the nonselective culture medium TSA was used, the CFU/g values of experimental group were statistically lower than the control group (Figure 1). These findings suggest that the antibiotic prophylaxis regimen being tested reduces the bacterial contamination of the bone particles harvested during the preparation of the implant site. A similar trend was found when the selective culture medium for streptococci isolation was used, although no significant differences in the CFU/g values were detected between the test and control groups. This finding may be due to the specific antibiotic prophylaxis regimen tested which consists of only two administrations.

The use of prophylactic antibiotics in implant dentistry is controversial. Esposito et al. [37], analyzing 6 studies included in a systematic review, suggested that 2 g or 3 g of amoxicillin given orally, as a single administration, one hour preoperatively significantly reduces failure of dental implants due to postoperative infection. Moreover, there is little research on the extent to which a single dose of amoxicillin reaches the surgical site and affects the oral microbiota, particularly the salivary bacteria that are responsible for the contamination of autogenous particulate bone graft [10, 11, 13, 17]. Larsson et al. [38] found that a single dose of amoxicillin, given as prophylaxis to prevent a surgical-site infection, results in a significant reducing effect on the oral streptococcal microflora in the gingival crevice, but not in saliva. The same authors were not able to detect amoxicillin in saliva at all time points after administration (1, 4, and 24 hours). On the other hand, Baglie et al. [39] reported the presence of measurable concentrations of amoxicillin in saliva until 8 hours after a single administration of a 875 mg tablet of amoxicillin. Moreover, amoxicillin was effective in reducing oral bacteria levels up to 12 hours' after administration.

## 5. Conclusions

This study showed that the antibiotic prophylaxis regimen tested (one 1 gr. tablet of amoxicillin plus clavulanic acid 12 hours and 1 hour before the surgery) reduces, but does not eliminate, bone particulate contamination. The most frequently isolated species belonged to the oral streptococci, but even pathogen species were detected. Further clinical research is needed to determine whether the antibiotic prophylaxis regimen tested is effective in minimizing infections after dental implant placement when an autogenous particulate bone graft is used.

## Figures and Tables

**Table 1:** Clinical features of test and control groups.

	Test group *N* = 18	Control group *N* = 16	*p* value
Age (Me (*Q*_1_–*Q*_3_))	53.5 (47.0–51)	50.0 (49.5–58.0)	0.261
Smoking status yes [*n* (%)]	5 (27.8)	5 (31.3)	0.824
Comorbidity yes [*n* (%)]	4 (22.2)	4 (25.0)	1.000

Me = median; *Q*_1_–*Q*_3_ = interquartile range.

**Table 2:** Detection frequency of bacterial species in test and control groups.

Species	Test group	Control group
*Streptococcus mitis*	38.8%	31.2%
*Streptococcus acidominimus*	33%	31.2%
*Streptococcus uberis*	22.2%	18.7%
*Streptococcus morbillorum*	16.6%	18.7%
*Streptococcus constellatus*	16.6%	0%
*Streptococcus mutans*	5.5%	6.25%
*Streptococcus oralis*	5.5%	0%
*Streptococcus sanguis*	5.5%	0%
*Streptococcus anginosus*	5.5%	0%
Coagulase-negative *Staphylococcus *	0%	12.5%
*Enterococcus faecalis*	11.1%	12.5%
*Lactobacillus salivarius*	0%	22.2%
*Streptococcus intermedius*	0%	12.5%
*Candida albicans*	0%	6.25%
*Gemella haemolysans*	0%	6.25%

**Table 3:** Microbiological findings in test and control groups.

Characteristics	Test group *N* = 18	Control group *N* = 16	*p* value
TSA log (Me (*Q*_1_–*Q*_3_))	1.71 (0.78–1.91)	2.12 (1.15–3.42)	0.018^*∗*^
MSA log (Me (*Q*_1_–*Q*_3_))	1.03 (0.60–1.78)	1.62 (0.82–3.03)	0.201
Bacterial species isolated (*n* (%)) 0 species 1 species 2 species 3 species 4 species	3 (16.7) 7 (38.9) 4 (22.2) 3 (16.7) 1 (5.6)	1 (6.3) 6 (37.5) 5 (31.3) 3 (18.8) 1 (6.3)	0.898

^*∗*^Statistically significant difference; Me = median; *Q*_1_–*Q*_3_ = interquartile range.

## References

[1] Buser D., Martin W., Belser U. C. (2004). Optimizing esthetics for implant restorations in the anterior maxilla: anatomic and surgical considerations. *The International Journal of Oral & Maxillofacial Implants*.

[2] Melilli D., Rallo A., Cassaro A. (2011). Implant overdentures: recommendations and analysis of the clinical benefits. *Minerva stomatologica*.

[3] Santos P. L., Gulinelli J. L., Telles Cda S., Betoni Jr. W., Okamoto R. (2013). Bone substitutes for peri-implant defects of postextraction implants. *International Journal of Biomaterials*.

[4] Melilli D., Mauceri N. (2015). Surface treatments for titanium implants. *International Journal of Clinical Dentistry*.

[5] Widmark G., Ivanoff C. J. (2000). Augmentation of exposed implant threads with autogenous bone chips: prospective clinical study.. *Clinical Implant Dentistry and Related Research*.

[6] Mayfield L. J. A., Skoglund A., Hising P., Lang N. P., Attström R. (2001). Evaluation following functional loading of titanium fixtures placed in ridges augmented by deproteinized bone mineral: a human case study. *Clinical Oral Implants Research*.

[7] Guerra I., Morais Branco F., Vasconcelos M., Afonso A., Figueiral H., Zita R. (2011). Evaluation of implant osseointegration with different regeneration techniques in the treatment of bone defects around implants: an experimental study in a rabbit model. *Clinical Oral Implants Research*.

[8] Sakkas A., Wilde F., Heufelder M., Winter K., Schramm A. (2017). Autogenous bone grafts in oral implantology—is it still a “gold standard”? a consecutive review of 279 patients with 456 clinical procedures. *International Journal of Implant Dentistry*.

[9] Esposito M., Grusovin M. G., Kwan S., Worthington H. V., Coulthard P. (2008). Interventions for replacing missing teeth: bone augmentation techniques for dental implant treatment. *Cochrane Database of Systematic Reviews*.

[10] Young M. P. J., Karachi M., Carter D. H., Worthington H., Drucker D. B. (2001). Microbial analysis of bone collected during implant surgery: a clinical and laboratory study. *Clinical Oral Implants Research*.

[11] Tezulas E., Dilek O. C. (2008). Decontamination of autogenous bone grafts collected from dental implant sites via osteotomy: a review. *Oral Surgery, Oral Medicine, Oral Pathology, Oral Radiology, and Endodontology*.

[12] Hashemi H. M., Beshkar M. (2011). Bacterial contamination of autogenous bone collected by rongeur compared with that collected by bone filter during implant surgery. *British Journal of Oral and Maxillofacial Surgery*.

[13] Takamoto M., Takechi M., Ohta K., Ninomiya Y., Ono S. (2013). Risk of bacterial contamination of bone harvesting devices used for autogenous bone graft in implant surgery. *Head & Face Medicine*.

[14] Esposito M., Hirsch J. M., Lekholm U., Thomsen P. (1998). Biological factors contributing to failures of osseointegrated oral implants: (II). Etiopathogenesis. *European Journal of Oral Sciences*.

[15] Young M. P. J., Korachi M., Carter D. H., Worthington H. V., McCord J. F. (2002). The effects of an immediately presurgical chlorhexidine oral rinse on the bacterial contaminants of bone debris collected during dental implant surgery. *Clinical Oral Implants Research*.

[16] Kürkçü M., Öz I. A., Köksal F., Benlidayi M. E., Güneşli A. (2005). Microbial analysis of the autogenous bone collected by bone filter during oral surgery: a clinical study. *Journal of Oral and Maxillofacial Surgery*.

[17] Koga T., Matsukubo T., Okuda K., Ishihara K. (2013). Effect of clinical factors on bacterial contamination of bone chips collected during implant surgery. *Implant Dentistry*.

[18] Blay A., Tunchel S., Sendyk W. R. (2003). Viability of autogenous bone grafts obtained by using bone collectors: histological and microbiological study. *Pesquisa Odontológica Brasileira*.

[19] Cawood J. I., Howell R. A. (1988). A classification of the edentulous jaws. *International Journal of Oral and Maxillofacial Surgery*.

[20] Olson M., O'Connor M., Schwartz M. L. (1984). Surgical wound infections. A 5-year prospective study of 20,193 wounds at the Minneapolis VA medical center. *Annals of Surgery*.

[21] Peterson L. J. (1990). Antibiotic prophylaxis against wound infections in oral and maxillofacial surgery. *Journal of Oral and Maxillofacial Surgery*.

[22] Buser D., Dula K., Belser U., Hirt H. P., Berthold H. (1993). Localized ridge augmentation using guided bone regeneration. 1. Surgical procedure in the maxilla. *The International Journal of Periodontics & Restorative Dentistry*.

[23] De Sanctis M., Zucchelli G., Clauser C. (1996). Bacterial colonization of bioabsorbable barrier material and periodontal regeneration. *Journal of Periodontology*.

[24] Nowzari H., London R., Slots J. (1995). The importance of periodontal pathogens in guided tissue regeneration and guided bone regeneration. *Compendium of Continuing Education in Dentistry*.

[25] Nowzari H., Slots J. (1995). Microbiologic and clinical study of polytetrafluoroethylene membranes for guided bone regeneration. *The International Journal of Oral & Maxillofacial Implants*.

[26] Yoshinari N., Tohya T., Mori A., Koide M., Kawase H. (1998). Inflammatory cell population and bacterial contamination of membranes used for guided tissue regenerative procedures. *Journal of Periodontology*.

[27] Graziani F., Cei S., Ivanovski S., La Ferla F., Gabriele M. (2007). A systematic review of the effectiveness of bone collectors. *The International Journal of Oral & Maxillofacial Implants*.

[28] Lambrecht J. T., Glaser B., Meyer J. (2006). Bacterial contamination of filtered intraoral bone chips. *International Journal of Oral and Maxillofacial Surgery*.

[29] Abu-Ta'a M., Quirynen M., Teughels W., Van Steenberghe D. (2008). Asepsis during periodontal surgery involving oral implants and the usefulness of peri-operative antibiotics: a prospective, randomized, controlled clinical trial. *Journal of Clinical Periodontology*.

[30] Anitua E., Aguirre J. J., Gorosabel A., Barrio P., Errazquin J. M. (2009). A multicentre placebo-controlled randomized clinical trial of antibiotic prophylaxis for placement of single dental implants. *European Journal of Oral Implantology*.

[31] Esposito M., Cannizzaro G., Bozzoli P., Consolo U., Felice P. (2008). Efficacy of prophylactic antibiotics for dental implants: a multicentre placebo-controlled randomized clinical trial. *European Journal Oral Implantol*.

[32] Esposito M., Cannizzaro G., Bozzoli P., Checchi L., Ferri V. (2010). Effectiveness of prophylactic antibiotics at placement of dental implants: a pragmatic multicenter placebo controlled randomized clinical trial. *European Journal of Oral Implantology*.

[33] Caiazzo A., Casavecchia P., Barone A., Brugnami F. (2011). A pilot study to determine the effectiveness of different amoxicillin regimens in implant surgery. *Journal of Oral Implantology*.

[34] Nolan R., Kemmoona M., Polyzois I., Claffey N. (2014). The influence of prophylactic antibiotic administration on post-operative morbidity in dental implant surgery. A prospective double blind randomized controlled clinical trial. *Clinical Oral Implants Research*.

[35] Chunduri N. S., Madasu K., Goteki V. R., Karpe T., Reddy H. (2012). Evaluation of bacterial spectrum of orofacial infections and their antibiotic susceptibility. *Annals of Maxillofacial Surgery*.

[36] Clarridge III J. E., Attorri S., Musher D. M., Hebert J., Dunbar S. (2001). Streptococcus intermedius, Streptococcus constellatus, and Streptococcus anginosus ("Streptococcus milleri group") are of different clinical importance and are not equally associated with abscess. *Clinical Infectious Diseases*.

[37] Esposito M., Grusovin M. G., Worthington H. V. (2013). Interventions for replacing missing teeth: antibiotics at dental implant placement to prevent complications. *Cochrane Database of Systematic Reviews*.

[38] Larsson W. C., Ryberg H., Sjöberg A. W. A., Blomqvist S., Colin P., Van Bocxlaer J. (2016). Antimicrobial effect of a single dose of amoxicillin on the oral microbiota. *Clinical Implant Dentistry and Related Research*.

[39] Baglie S., Del Ruenis A. P., Motta R. H., Baglie R. C., Franco G. C., Franco L. M. (2007). Plasma and salivary amoxicillin concentrations and effect against oral microorganisms. *International Journal of Clinical Pharmacology and Therapeutics*.

